# A Statistical Model of Cleavage Fracture Toughness of Ferritic Steel DIN 22NiMoCr37 at Different Temperatures

**DOI:** 10.3390/ma12060982

**Published:** 2019-03-25

**Authors:** Guian Qian, Wei-Sheng Lei, Zhenfeng Tong, Zhishui Yu

**Affiliations:** 1 State Key Laboratory of Nonlinear Mechanics (LNM), Institute of Mechanics, Chinese Academy of Sciences, Beijing 100190, China; qianguian@imech.ac.cn; 2 Applied Materials, Inc., 974 East Arques Avenue, Sunnyvale, CA 94085, USA; 3 China Institute of Atomic Energy, Beijing 102413, China; 4 School of Materials Engineering, Shanghai University of Engineering Science, 333 Long Teng Rd., Shanghai 201620, China; yu_zhishui@163.com

**Keywords:** statistical model, ferritic steels, cleavage fracture toughness, master curve behavior

## Abstract

It is a conventional practice to adopt Weibull statistics with a modulus of 4 for characterizing the statistical distribution of cleavage fracture toughness of ferritic steels, albeit based on a rather weak physical justification. In this study, a statistical model for cleavage fracture toughness of ferritic steels is proposed according to a new local approach model. The model suggests that there exists a unique correlation of the cumulative failure probability, fracture toughness and yield strength. This correlation is validated by the Euro fracture toughness dataset for 1CT specimens at four different temperatures, which deviates from the Weibull statistical model with a modulus of four.

## 1. Introduction

Recently there have been a lot of studies on fatigue mechanism and modeling of fracture behavior of engineering materials using multiscale and probabilistic approaches [1,2,3,4,5,6,7,8,9]. During the long-term service of structures, the structural integrity should be performed using different methods, e.g., deterministic and probabilistic methods [1,2,3,4,5,6,7]. The different ageing mechanism, e.g., fracture [1,4,6], fatigue [2,3,5,7], and failure modes should also be studied and advanced crack detection setups developed [8]. In this paper, fracture is considered as the ageing mechanism.

Ferritic steels are commonly used for fabricating nuclear reactor pressure vessels of extremely stringent structural integrity requirement. However, owing to their body center cubic crystalline structures, ferritic steels are susceptible to cleavage fracture. The random distribution of carbides and other cleavage nuclei in steels causes large dispersion and significant size effect of cleavage fracture toughness, which calls for a statistical approach to cleavage fracture toughness assessment Lei [9] presented a comprehensive critical review on the statistical models of cleavage fracture toughness. In brief, among numerous studies, the following four major approaches are noteworthy:

- The empirical description of cleavage fracture toughness using Weibull statistics pioneered by Landes and colleagues [10,11,12].
- The Beremin model [13,14].
- The “Master Curve” method [15,16,17].
- The Prometey Unified Curve model [16,18,19].

As a common feature of these four approaches, Weibull statistics is commonly adopted for cleavage fracture toughness distribution. Initially, Weibull statistics was used for empirical description [8,9,10,11,12]. Later on, the other three approaches concluded that Weibull distribution of cleavage fracture toughness is a derivative of the local approach to cleavage fracture [13,14,15,16,17,18,19].

Landes and co-workers [10,11,12] adopted the Weibull statistics to fit cleavage fracture toughness data in terms of $J_{c}$, the critical *J*-integral at cleavage fracture, or $K_{Jc}$, the critical stress intensity factor at cleavage fracture, as follows

(1) $$P=1-exp\left[-{\left(\frac{J_{C}-J_{min}}{J_{0}}\right)}^{m_{J}}\right]$$

(2) $$P=1-exp\left[-{\left(\frac{K_{Jc}-K_{min}}{K_{0}}\right)}^{m_{K}}\right]$$

where *P* is failure probability, *J*_min_ and *K*_min_ are the threshold values, *J*_0_ and *K*_0_ are scale parameters, *m_J_* and *m_K_* are Weibull modulus. Note that he probabilities in Equations (1) and (2) are the same but only in terms of different parameters. When the two-parameter Weibull model ($J_{min}=K_{min}=0$) was adopted, $m_{J}\approx 5$ or $m_{K}\approx 10$ was obtained in [10]; while with the three-parameter Weibull model, $m_{K}=0.9–4.7$ and $K_{min}=0–109\;\mathrm{MPa}\sqrt{m}$ were obtained for different data sets [11,12]. Equations (1) and (2) are purely empirical and in fact conflict to each other. As analyzed in detail in Reference [20], due to the following relationship between $K_{Jc}$ and $J_{c}$,

(3) $$K_{Jc}=\sqrt{EJ_{c}/\left(1-\nu^{2}\right)}$$

where the Weibull failure probabilities take the form of

(4) $$P=1-exp\left[-{\left(\frac{J_{C}-J_{min}}{J_{0}}\right)}^{m_{J}}\right]\Rightarrow P=1-exp\left[-{\left(\frac{K_{Jc}^{2}-K_{min}^{2}}{K_{0}^{2}}\right)}^{m_{J}}\right]$$

(5) $$P=1-exp\left[-{\left(\frac{K_{Jc}-K_{min}}{K_{0}}\right)}^{m_{K}}\right]\Rightarrow P=1-exp\left[-{\left(\frac{\sqrt{J_{c}}-\sqrt{J_{min}}}{\sqrt{J_{0}}}\right)}^{m_{K}}\right]$$

Equations (4) and (5) indicate that if $J_{c}$ is described by a three-parameter Weibull distribution, the quantity $K_{Jc}^{2}$, instead of $K_{Jc}$, will follow the same distribution; while if $K_{Jc}$ is described by a three-parameter Weibull distribution, the quantity $\sqrt{J_{c}}$, instead of $J_{c}$, will follow the same distribution. Only when both $K_{Jc}$ and $J_{c}$ are described by two-parameter Weibull statistics, there is $m_{K}=2m_{J}$ via the following relationship:

(6) $$P=1-exp\left[-{\left(\frac{J_{C}}{J_{0}}\right)}^{m_{J}}\right]\Rightarrow P=1-exp\left[-{\left(\frac{K_{Jc}^{2}}{K_{0}^{2}}\right)}^{m_{J}}\right]=1-exp\left[-{\left(\frac{K_{Jc}}{K_{0}}\right)}^{m_{K}}\right]$$

In view of the shortcomings in using the empirical Weibull distributions in Equations (1) and (2) for cleavage fracture toughness characterization, there is a necessity to rationalize a cleavage fracture toughness model by pursuing a statistical approach to the inherently random occurrence of cleavage fracture and considering the fundamental role of cleavage mechanisms. The Beremin model was proposed as below [13,14]:

(7) $$P=1-\exp\left[-\left(\int_{V_{pl}}\sigma_{1}^{m}dV/V_{0}\right)/\sigma_{0}^{m}\right]=1-\exp\left[-{\left(\sigma_{W}/\sigma_{0}\right)}^{m}\right]$$

(8) $$\sigma_{W}={\left(\int_{V_{pl}}\sigma_{1}^{m}dV/V_{0}\right)}^{1/m}$$

where *m* is Weibull modulus, σ_0_ is scale parameter, $\sigma_{W}$ is the Weibull stress, *V_pl_* is the volume of plastic deformation zone, *V*_0_ is the mean volume occupied by each micro-crack, *σ*_1_ is the maximum tensile principal stress. Specific to small scale yielding for a mode I crack problem, Equation (7) led to the two-parameter Weibull distribution of *K_Ic_* with a modulus of 4 [13,14]:

(9) $$P=1-\exp\left(-\frac{B\cdot K_{Jc}^{4}\cdot \sigma_{ys}^{m-4}\cdot C_{m,n}}{V_{0}\cdot \sigma_{0}^{m}}\right)=1-exp\left[-{\left(\frac{K_{Jc}}{K_{0}}\right)}^{4}\right]$$

(10) $$K_{0}={\left(\frac{V_{0}\sigma_{0}^{m}}{C_{m,n}B\sigma_{ys}^{m-4}}\right)}^{1/4}$$

Here *C_m,n_* is a numerical coefficient, *σ_ys_* is yield stress, *B* is specimen thickness.

For large scale yielding, the situation becomes more complex. Equation (2) is needed to calculate the Weibull stress $\sigma_{W}$ at each *K_Jc_* so as to establish the $\sigma_{W}-P\;$and${\;K}_{Jc}-P$ correlations numerically.

**Figure 1:**
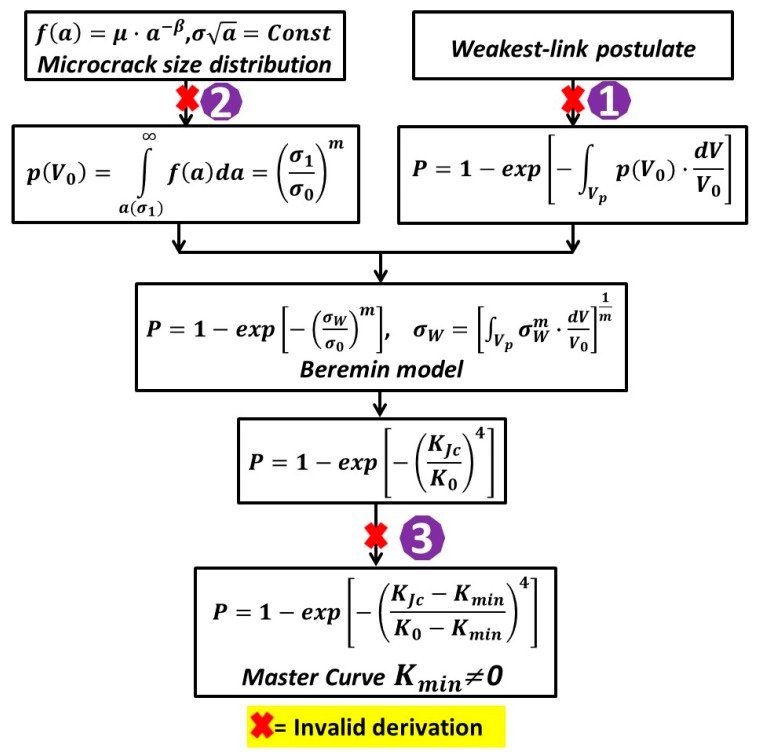
Formulation process of Beremin model and the non-transferrability of two- and three- parameter Weibull statistics of fracture toughness [9,21,22,23,24].

As highlighted in Figure 1 [21,22,23,24], the Beremin model suffers from some fundamental defects, leading to the invalidity of Equations (7) and (9). The necessary corrections to the Beremin model are also provided to ensure the mathematical rigorousness and the physical compliance with the five assumptions below: (1) The uniform spatial distribution of microcracks, (2) the weakest-link postulate of brittle fracture, (3) plastic yielding as a prerequisite for cleavage fracture, (4) the maximum tensile principal stress criterion for cleavage fracture, (5) the power-law distribution of microcrack size. In Figure 1, the weakest link concept is not strictly followed as the above five assumptions are not met. Derivation 1 and 2 are not valid because that Assumption 2 and 3 are not met. Derivation 3 is not valid because assumption 3 is not met.

Using a methodology same as the Beremin model but with a more detailed analysis, Wallin and co-workers [15,16,17] obtained the two-parameter Weibull model of fracture toughness that is essentially the same as Equation (9). Considering that cleavage fracture should not occur at infinitesimal loading, in the lack of any rigorous mathematical deduction, a threshold $K_{min}$ was arbitrarily introduced to rewrite Equation (9) as below with a given thickness at a given temperature [15,16,17]:

(11) $$P=1-exp\left[-{\left(\frac{K_{Jc}-K_{min}}{K_{0}-K_{min}}\right)}^{4}\right]$$

Equation (11) is usually called the Master Curve model for cleavage fracture toughness statistics. The ASTM E 1921-11 testing standard sets $K_{min}=20\;\mathrm{MPa}\sqrt{m}$ for all ferritic steels.

Since Equation (11) is based on the two-parameter Weibull model Equation (9), it lacks a strict physical justification. A more detailed dissection of the model proposed by Wallin and co-workers [15,16,17] is reported in [9]. The Prometey approach [16,18,19] includes two statistical models for cleavage fracture. The simplified model assumes that only the microcrack nucleation resistance σ_d_ is taken as a stochastic parameter while the microcrack propagation resistance *S*(ε*_p_*) as a function of local plastic strain ε*_p_* is a deterministic one. The comprehensive model assumes both the microcrack nucleation resistance σ*_d_* and the microcrack propagation resistance *S*(ε*_p_*) as stochastic parameters. This approach led to a theoretical model of fracture toughness essentially identical to the Beremin model Equation (9), although their experimental results fit better to the general two-parameter Weibull model in Equation (2) with $m_{K}=4–30$ [18].

It is interesting to note that all the three models fall into the local approach methodology which is based on the weakest link postulate and the understanding of the dominant microscopic cleavage fracture mechanisms. But they all land on a two- or three- parameter Weibull statistics with a modulus (*m_K_*) of 4 and fixed-value threshold (*K_min_*) to describe the statistical distribution of cleavage fracture toughness (*K_Ic_*). The minor difference is that $K_{min}=0$ for the Beremin model, while $K_{min}=20\;\mathrm{MPa}\sqrt{m}$ in the Master Curve approach and $K_{min}=26\;\mathrm{MPa}\sqrt{m}$ in the Prometey Unified Curve model.

In a series of previous work [9,21,22,23], it was revealed that the prevailing adoption of two- or three-parameter Weibull distribution of cleavage fracture toughness with a modulus of 4 and a fixed-value threshold independent of temperature and plastic constraint lacks a solid theoretical foundation. Consequently, a new local approach model of cleavage fracture was proposed. This work will characterize the statistics of cleavage fracture toughness based on a new local approach model of cleavage fracture [9,21,22,23,24,25].

This paper aims to statistically characterize cleavage fracture toughness of DIN 22NiMoCr37 according to a new local approach and develop a new “Master Curve” to describe the cleavage fracture behavior using the local approach and probabilistic concepts.

## 2. A Statistical Model of Cleavage Fracture Toughness

According to the new local approach model for brittle fracture in References [9,21,22,23,24,25], the cumulative failure probability is formulated as follows for a uniform spatial distribution of microcracks:

(12) $$P=1-\exp\left\{\int_{V_{pl}}\ln\left[1-p(V_{0})\right]\cdot dV/V_{0}\right\}$$

where *p*(*V*_0_) is the fracture probability of an elementary volume (*V*_0_) induced by an embedded microcrack under an arbitrary stress state. Under the maximum tensile stress fracture criterion, when *g*(*S*), the probability density function of the microscopic cleavage fracture strength (S), takes the three-parameter Weibull distribution,

(13) $$g\left(S\right)=\frac{m}{\sigma_{0}}\cdot {\left(\frac{S-\sigma_{th}}{\sigma_{0}}\right)}^{m-1}\cdot \exp\left[-{\left(\frac{S-\sigma_{th}}{\sigma_{0}}\right)}^{m}\right]$$

we get,

(14) $$p\left(V_{0}\right)=\int_{\sigma_{1,0}}^{\sigma_{1}}g\left(S\right)dS=1-exp\left[-{\left(\frac{\sigma_{1}-\sigma_{1,0}}{\sigma_{0}}\right)}^{m}\right]$$

(15) $$P=1-\exp\left\{-\int_{V_{pl}}\left[{\left(\sigma_{1}-\sigma_{1,0}\right)}^{m}/\sigma_{0}^{m}\right]\cdot dV/V_{0}\right\}=1-\exp\left[-{\left(\sigma_{W}/\sigma_{0}\right)}^{m}\right]$$

where $\sigma_{1,0}$ is the maximum principal stress at initial yielding of a differential volume element dV, $\sigma_{th}$ is threshold stress and $\sigma_{th}\;\mathrm{is}\;\sigma_{1,0}$ to observe the precedence of plastic yielding over cleavage fracture [9,21,22,23,24,25]. Note that in Equation (15), the following new definition of Weibull stress $\sigma_{W}$ is introduced

(16) $$\sigma_{W}={\left(\int_{V_{pl}}{\left(\sigma_{1}-\sigma_{1,0}\right)}^{m}dV/V_{0}\right)}^{1/m}$$

Equation (16) suggests that the new Weibull stress $\sigma_{W}$ is affected by material constitutive properties, specimen constraint effect, Weibull modulus *m*, and external load. The new local approach has been validated for the statistical assessment of cleavage fracture in notched specimens [24,25,26]. For a notched specimen at a fixed temperature (*T*), the new Weibull stress $\sigma_{W}$ only vary with m and the nominal stress $\sigma_{N}$ as that is,

(17) $$\sigma_{W}\left(T=T_{1}\right)=f\left(m,\sigma_{N}\right)\;$$

Since Weibull modulus *m* is assumed as a temperature-independent material property, while the yield stress $\sigma_{ys}$ strongly depends on temperature, Equation (17) is rewritten as [24,25,26] to consider temperature effect:

(18) $$\frac{\sigma_{W}}{\sigma_{ys}}=\frac{\sigma_{W}\left(T\right)}{\sigma_{ys}\left(T\right)}=f\left(\frac{\sigma_{N}\left(T\right)}{\sigma_{ys}\left(T\right)}\right)\;$$

Equation (18) suggests that there exists a unique correlation (sometimes also called a “master curve”) between the two normalized variables $\frac{\sigma_{W}}{\sigma_{ys}}$ and $\frac{\sigma_{N}}{\sigma_{ys}}$ at different temperatures. While it can be difficult to obtain the analytical expression of function y=f(x), it is expected that f(x) is a non-linear function and depends on specific notch geometry and loading mode. Substitution of Equation (18) in Equation (15) leads to

(19) $$P=1-exp\left[-{\left(\frac{\sigma_{ys}}{\sigma_{0}}\right)}^{m}f^{m}\left(\frac{\sigma_{N}}{\sigma_{ys}}\right)\right]$$

Or

(20) $$Y=\frac{1}{\sigma_{ys}}\cdot {\left\{ln\left[\frac{1}{\left(1-P\right)}\right]\right\}}^{\frac{1}{m}}=\frac{1}{\sigma_{0}}\cdot f\left(\frac{\sigma_{N}}{\sigma_{ys}}\right)$$

The studies [23,24,25,26] for both side edge notched prismatic specimens in bending and circumferentially notched round specimens in tension have validated the expected correlation between the compound parameters $Y=\frac{1}{\sigma_{ys}}\cdot {\left\{ln\left[\frac{1}{\left(1-P\right)}\right]\right\}}^{\frac{1}{m}}$ and $X=\frac{\sigma_{N}}{\sigma_{ys}}$ at different temperatures and the temperature indepdence of Weibull modulus m.

Now in this study, we further extend the new local approach model in Equation (15) to the case of a pre-cracked specimen for evaluating cleavage fracture toughness. Equation (15) is rewritten as

(21) $$P=1-exp\left[-{\left(\frac{\sigma_{ys}}{\sigma_{0}}\right)}^{m}\cdot \left(\frac{V_{pl}}{V_{0}}\right)\cdot \int_{V_{pl}}^{}{\left(\frac{\sigma_{1}-\sigma_{1,0}}{\sigma_{ys}}\right)}^{m}\cdot \frac{dV}{V_{pl}}\right]=1-exp\left[-{\left(\frac{\sigma_{ys}}{\sigma_{0}}\right)}^{m}\cdot \left(\frac{V_{pl}}{V_{0}}\right)\cdot \phi \left(m\right)\right]$$

(22) $$\phi \left(m\right)=\int_{V_{pl}}{\left(\frac{\sigma_{1}-\sigma_{1,0}}{\sigma_{ys}}\right)}^{m}\cdot \frac{dV}{V_{pl}}$$

Note that the compound parameter $B{\left(K_{Jc}/\sigma_{ys}\right)}^{4}$ has the same dimension as the volume $V_{pl}$. According to fracture mechanics, under small scale yielding, the following direct proportion exists,

(23) $$V_{pl}\propto B{\left(K_{Jc}/\sigma_{ys}\right)}^{4}$$

However, under large scale yielding, Equation (23) is no longer valid. However, dimensional consistency permits to express $V_{pl}$ as a function of $B{\left(K_{Jc}/\sigma_{ys}\right)}^{4}$,

(24) $$\frac{V_{pl}}{V_{0}}=f\left(B{\left(K_{Jc}/\sigma_{ys}\right)}^{4}/V_{0}\right)$$

According to Equation (24), so long as there is a non-linear correlation between $V_{pl}\;$and$\;B{\left(K_{Jc}/\sigma_{ys}\right)}^{4}$, the Weibull statistics of fracture toughness *K_Jc_* with a fixed modulus of 4 does not exist.

Substitution of Equation (24) in Equation (21) yields

(25) $$P=1-exp\left[-{\left(\frac{\sigma_{ys}}{\sigma_{0}}\right)}^{m}\cdot f\left(B{\left(K_{Jc}/\sigma_{ys}\right)}^{4}/V_{0}\right)\cdot \phi \left(m\right)\right]$$

Or

(26) $$\sqrt[{m}]{Ln\left[\frac{1}{\left(1-P\right)}\right]}/\sigma_{ys}=\left[\sqrt[{m}]{\phi \left(m\right)}/\sigma_{0}\right]\cdot \sqrt[{m}]{f\left(B{\left(K_{Jc}/\sigma_{ys}\right)}^{4}/V_{0}\right)}$$

Equation (26) suggests that there is an inherent correlation between $\sqrt[{m}]{Ln\left[\frac{1}{\left(1-P\right)}\right]}/\sigma_{ys}$ and $B{\left(K_{Jc}/\sigma_{ys}\right)}^{4}$.

By now, the work derives a physical correlation between the two compound parameters $\sqrt[{m}]{Ln\left[\frac{1}{\left(1-P\right)}\right]}/\sigma_{ys}$ and $B{\left(K_{Jc}/\sigma_{ys}\right)}^{4}$ prior to the calibration of Weibull parameters (*m*, *σ*_0_) and ϕ(m) in Equation (22). The method to calibrate Equation (15) has been developed in [23,24,25] for notched specimens for determination of m and *σ*_0_, and is under evaluation for fracture mechanics specimens based on Equation (21) to obtain the values of m, *σ*_0_ and ϕ(m). Once Equation (21) is calibrated, the expression of $f\left(B{\left(K_{Jc}/\sigma_{ys}\right)}^{4}/V_{0}\right)$ will become explicit. Detailed numerical analysis is ongoing to determine the value of ϕ(m) and the expression of Equation (24) for fracture toughness specimens of different crack size and at different temperatures. However, it is of immediate interest to first justify and validate Equation (24) under large scale yielding. In the following, Equation (26) is used to analyze a group of published fracture toughness data. 

## 3. Model Validation

**Figure 2:**
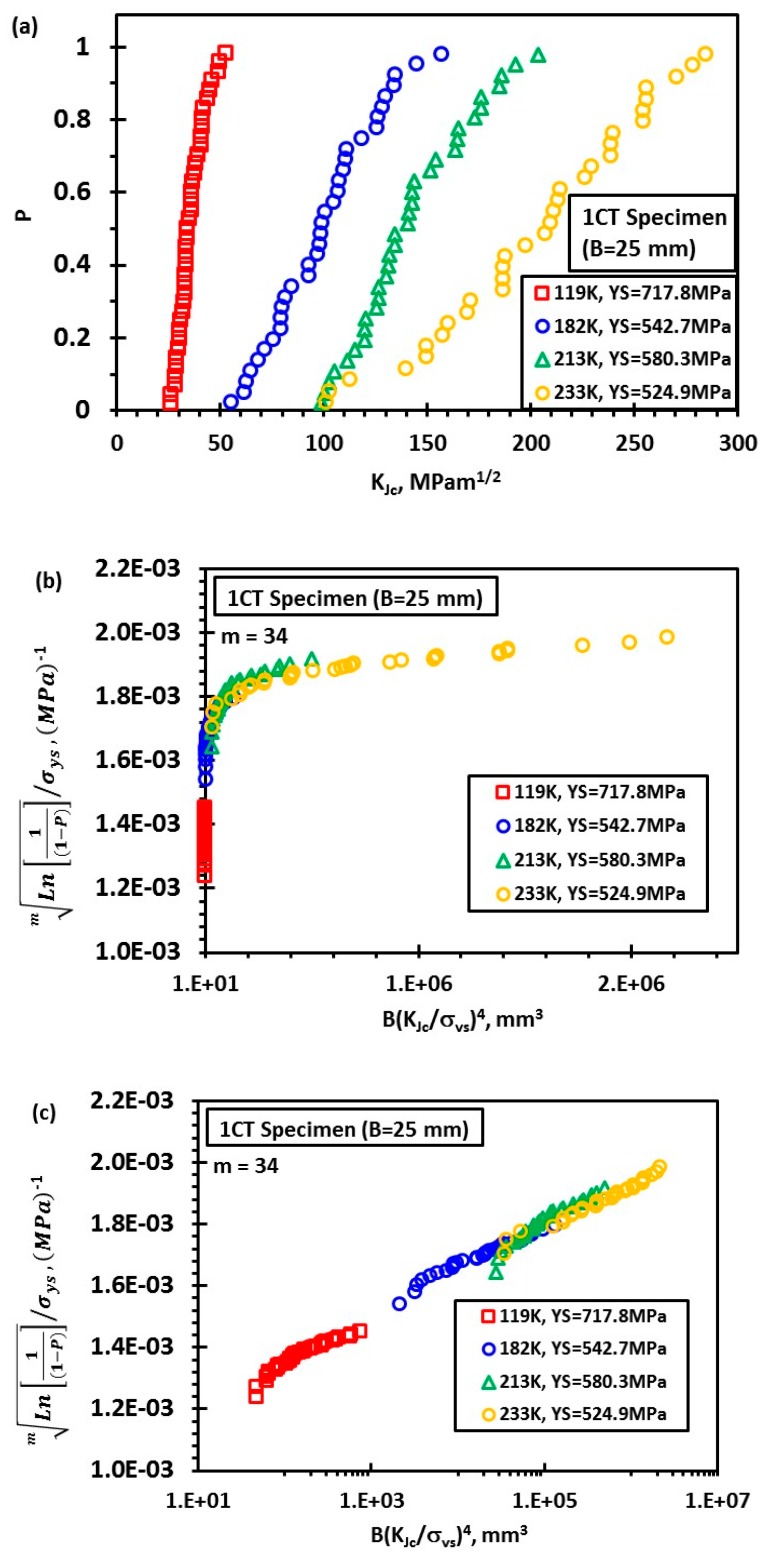
Fracture toughness of steel DIN 22NiMoCr37 measured by 1CT specimens: Raw data [27] (**a**); the correlation between $\sqrt[{m}]{Ln\left[\frac{1}{\left(1-P\right)}\right]}/\sigma_{ys}$ and $B{\left(K_{Jc}/\sigma_{ys}\right)}^{4}$ with *x*-axis in linear scale (**b**); and logarithmic scale (**c**).

**Figure 3:**
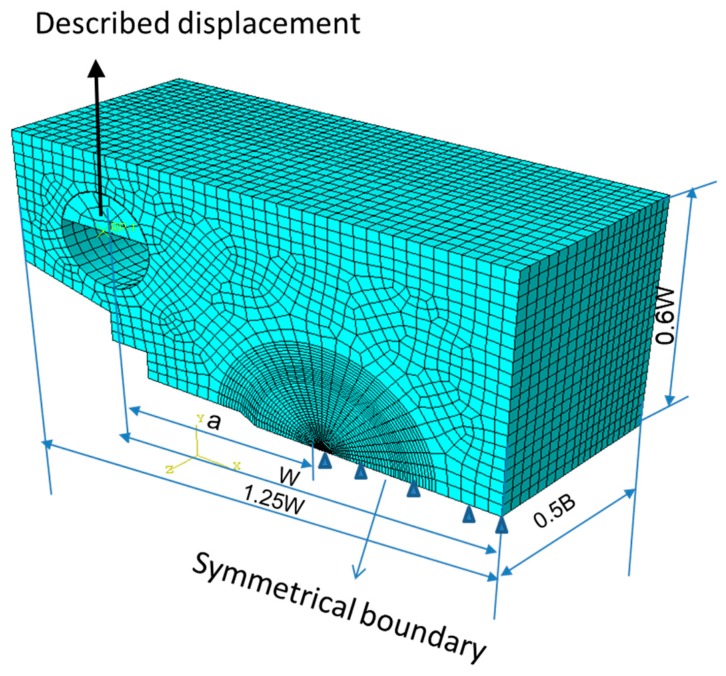
Finite element meshes of a half-sized 1CT specimen.

**Figure 4:**
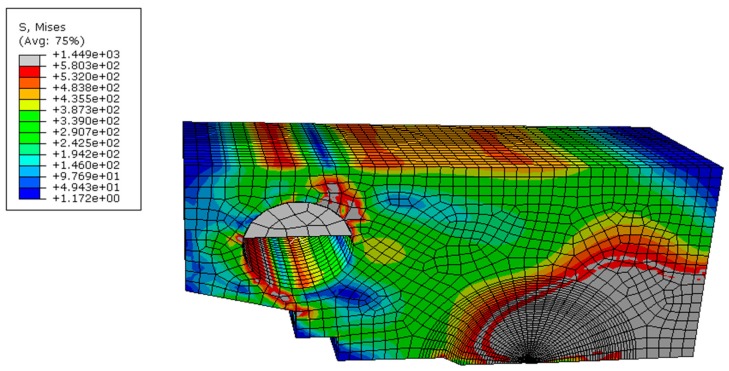
An example of stress distribution in 1CT specimen at 182 K.

**Figure 5:**
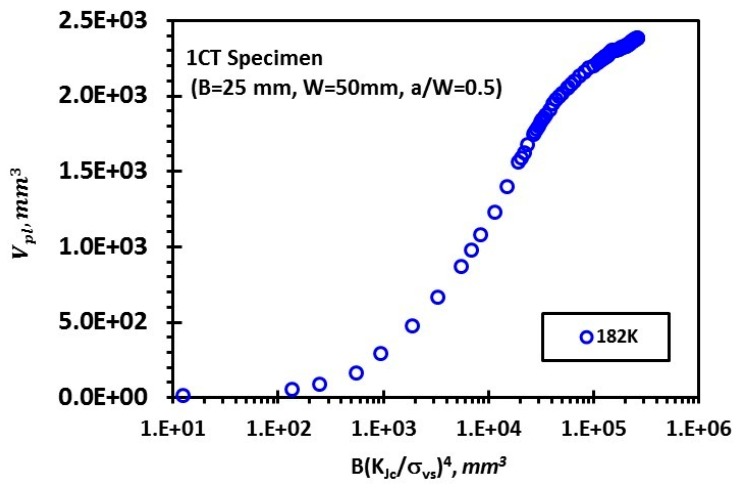
Variation of volume of plastic zone *V*_pl_ with the compound parameter *B*(*K_Jc_*/*σ_ys_*)^4^.

The Euro fracture toughness dataset summarizes the fracture behavior of the quenched and tempered pressure vessel steel DIN 22NiMoCr37 with about 800 fracture toughness tests on 1/2T to 4T CT-specimens. The dataset is available at the address ftp://ftp.gkss.de/pub/eurodataset. Heerens and Hellmann [27] provided its essential background information. Figure 2a shows the rank probability vs. fracture toughness data of 1CT specimens (B = 25 mm) at four different temperatures with yield strength (YS) also provided as follows: YS = 717.8 MPa at 119 K, 580.2 MPa at 182 K, 542.7 MPa at 213 K, and 524.9 MPa at 233 K. The toughness data of a total number of N at each temperature were ranked in an ascending order and the *i*-th datum was assigned with a rank probability $P_{i}=\left(i-0.3\right)/\left(N+0.4\right)$, *i* = 1, 2…, *N*. Figure 2b,c shows the correlation between $\sqrt[{m}]{Ln\left[\frac{1}{\left(1-P\right)}\right]}/\sigma_{ys}$ and $B{\left(K_{Jc}/\sigma_{ys}\right)}^{4}$ at four temperatures for *m* = 34 with axis in linear scale and logarithmic scale, respectively. Obviously, all the data fall onto a master curve described by certain nonlinear relationship between $\sqrt[{m}]{Ln\left[\frac{1}{\left(1-P\right)}\right]}/\sigma_{ys}$ and $B{\left(K_{Jc}/\sigma_{ys}\right)}^{4}$. By now, the analysis is purely based on Equation (26). In order to validate the non-linear dependence of$\;V_{pl}$ on the compound parameter $B{\left(K_{Jc}/\sigma_{ys}\right)}^{4}$ in Equation (24), finite element analysis of 1CT specimen with thickness *B* = 25 mm, width *W* = 50 mm, and the crack depth *a* to specimen width *W* ratio *a*/*W* = 0.5, was conducted at 182 K using ABAQUS 6.14 (Figure 3) with *E =* 206 GPa, *ν* = 0.3, and the plastic behavior of the material as reported in [27]. Due to symmetry considerations, only one half of the specimen was modeled. The mesh and model are shown in Figure 3. The displacement was applied on a rigid pin in frictionless contact with specimen and the applied load was obtained from the reaction force acting on the rigid body. A finite strain (large deformation theory) method is used. A total of 5046120-node brick elements was used. The J-integral was computed using the domain integral implemented in ABAQUS 6.14, which calculates the J-integral over a predefined number of contours around the crack tip. Then the J-integral was converted to the stress intensity factor $K_{J}=\sqrt{JE/\left(1-\nu^{2}\right)}$ under plane strain condition. The Weibull stress *σ_w_* for a certain stress is determined from a post processing program, which reads the ABAQUS output file. The volume of the cleavage fracture process zone *V*, *K*_J_ and the principal stress σ_1_ of each node were obtained at each time step. The volume of plastic zone *V_pl_* at a certain stress (*K_J_* or *J*) was determined from a post processing program, which reads the ABAQUS output file. *V_pl_* was calculated at each time step according to the stresses on Gauss points of the elements, which takes the weight of the Gauss points in the integration. Figure 4 shows an example of stress distribution inside the specimen. Figure 5 summarizes the calculated volume of plastic deformation zone $V_{pl}$ under different loading level represented by the compound parameter $B{\left(K_{Jc}/\sigma_{ys}\right)}^{4}$. It clearly reveals the non-linear relationship between $V_{pl}$ and $B{\left(K_{Jc}/\sigma_{ys}\right)}^{4}$.

The purpose of this work is to present a theoretical justification summarized as Equation (26) to guide statistical characterization of cleavage fracture toughness. For all fracture toughness data measured from different sized specimens (with thickness of 12.5 mm, 25 mm, 50 mm, 100 mm), according to Equation (26), finite element calculations will be arranged to precisely determine the value of ϕ(m) defined by Equation (22) and the function $f\left(B{\left(K_{Jc}/\sigma_{ys}\right)}^{4}/V_{0}\right)$ defined by Equation (24) at each thickness. It is noted that this paper focuses on the discussion of ferritic steels for nuclear reactor pressure vessel. Although the specific steel types used in different countries are different, the structure and mechanical properties are basically similar. The steel used in this paper are the most important (dominant) in the nuclear industry. Thus, they are selected as the research steel for European Union Round Robin project by ESIS. For other ferritic structural steels such as railway bridges, specific loading and service conditions such as dynamic loading and cyclic loading should be considered for specific research. These large-scale fracture toughness tests for nuclear power steels at low temperatures are very expensive. Thanks to the European Union project, the authority of the data is proved. These data are obtained by ESIS for the round robin project of nuclear power, which is participated by 15 research institutes of European countries. They include more than 800 fracture toughness data measured by CT samples of different sizes. These data are highly recommended by the industry. Different authors have analyzed them from different perspectives. We study them from a new perspective. A lot of finite element analysis for the specimens with different sizes is under way. This paper introduces the results of 1CT specimen at different temperatures. Further results will be reported soon.

Further work on finite element analysis of the volume of plastic deformation zone in other different sized fracture toughness specimens including 1/2CT, 2CT, and 4CT is ongoing and will be reported separately.

## 4. Conclusions

1. A model for the statistical distribution of cleavage fracture toughness is proposed based on a new local approach model to collectively reflect the effect of temperature and specimen size. The model suggests that under large scale yielding, the distribution of cleavage fracture toughness may deviate from the Weibull statistics with a modulus (m_K_) of four.
2. According to the proposed model, cleavage fracture toughness data of 1CT specimens at four different temperatures are synchronized onto a single master curve governed by the two compound parameters $\sqrt[{m}]{Ln\left[\frac{1}{\left(1-P\right)}\right]}/\sigma_{ys}$ and $B{\left(K_{Jc}/\sigma_{ys}\right)}^{4}$.
3. Finite element analysis of stress distribution in a 1CT fracture toughness specimen reveals the non-linear relationship between $V_{pl}$ and $B{\left(K_{Jc}/\sigma_{ys}\right)}^{4}$ under large scale yielding.

It should be noted that the probabilistic method developed in this paper will be used in developing a framework of fatigue lifetime prediction and fatigue study of engineering structures [28,29,30]. Biaxial effect should also be considered in developing the probabilistic framework [4,31].

## Nomenclature

*a*	crack depth
*B*, *W*, *L*	geometrical dimensions of a specimen
*C_m,n_*	numerical coefficient
*CT*	compact tension
*E*	Young’s modulus, MPa
*f*(*a*)	probability density function of microcrack size (a) distribution
*g*(*S*)	probability density function of microscopic cleavage strength (*S*)
*J*	J-integral
*J*_c_	critical J-integral at cleavage fracture
*j*	rank number
*J_min_*, *K_min_*	threshold values
*J*_0_, *K*_0_	scale parameters
*K*	stress intensity factor
$K_{Jc}$	critical stress intensity factor at cleavage fracture
*m, m_J_, m_K_*	Weibull modulus
*P*	fracture probability
*p*(*V*_0_)	fracture probability of an elementary volume (*V*_0_)
S	fracture strength
*S*	microscopic cleavage fracture strength
*S*(ε*_p_*)	microcrack propagation resistance
*V_pl_*	volume of plastic deformation zone
*V*_0_	mean volume occupied by each micro-crack
*dV*	differential volume
ΔV	volume of a finite element
*ν*	Poisson’s ratio
*σ_ys_*	yield stress
*σ*_0_	scale parameter
*σw*	Weibull stress
*σ*_1_	maximum tensile principal stress
$\sigma_{1,0}$	maximum principal stress at initial yielding of a volume dV
$\sigma_{th}$	threshold stress
*σ*_d_	resistance to microcrack nucleation
ε_p_	plastic strain
ϕ(m)	numerical coefficient
